# Microstructural and Process Characterization of Conductive Traces Printed from Ag Particulate Inks

**DOI:** 10.3390/ma4060963

**Published:** 2011-05-26

**Authors:** David A. Roberson, Ryan B. Wicker, Lawrence E. Murr, Ken Church, Eric MacDonald

**Affiliations:** 1 Department of Metallurgical and Materials Engineering, The University of Texas at El Paso, El Paso, TX 79968, USA; E-Mail: lemurr@utep.edu; 2 W. M. Keck Center for 3D Innovation, The University of Texas at El Paso, El Paso, TX 79968, USA; E-Mails: rwicker@utep.edu (R.B.W.); khchurch@utep.edu (K.C.); emac@utep.edu (E.M.); 3 Department of Mechanical Engineering, The University of Texas at El Paso, El Paso, TX 79968, USA; 4 Department of Electrical and Computer Engineering, The University of Texas at El Paso, El Paso, TX 79968, USA

**Keywords:** conductive ink, Ag nanoparticles, inkjet, scanning electron microscopy, microstructures

## Abstract

Conductive inks are key enablers for the use of printing techniques in the fabrication of electronic systems. Focus on the understanding of aspects controlling the electrical performance of conductive ink is paramount. A comparison was made between microparticle Ag inks and an Ag nanoparticle ink. The microstructures resulting from thermal cure processes were characterized morphologically and also in terms of their effect on the resistivity of printed traces. For microparticle inks, the variability of resistivity measurements between samples as defined by coefficient of variation (CV) was greater than 0.1 when the resistivity was 10 to 50 times that of bulk Ag. When the resistivity was lower (~1.4 times that of bulk Ag) the CV of sample sets was less than 0.1. In the case of the nanoparticle ink, resistivity was found to decrease by a factor ranging from 1.2 to 1.5 after doubling the amount of layers printed prior to curing though it was expected to remain the same. Increasing the amount of layers printed also enhanced the sintering process.

## 1. Introduction

The creation of conductive paths in electronic systems has traditionally involved the addition and subtraction of conductive material by means of deposition, mask, and etch processing [1,2]. Printing techniques, such as rotogravure, screen printing, inkjet, and direct write micro dispense have been proven as viable alternative methods for creating conductive paths and have the additional benefit of creating these paths in a simple two step process involving only printing and curing [3,4,5,6]. Conductor loaded inks allow printing technologies to be applied in the creation of electronic systems. Beyond the creation of conductive paths for use as interconnects, conductive inks have been utilized in the creation of electronic components such as transistors, RF antennas, and glucose sensors [7,8,9] among others.

The thermal cure process produces microstructural changes in particle-based conductive inks (of both the micro and nano scale), which impacts the overall electrical properties of a printed conductive trace. In the case of inks where the particle size is of the micron scale, low temperature curing (between 100 °C and 200 °C for inks designed to work in this temperature range) in which the amount of material suspending the conductive particles will decrease, resulting in particles conducting through physical contact in a process known as percolation [3,10,11,12]. This mechanism has been demonstrated by Saraf *et al.* [12] for an Ag microflake conductive ink. In [12], a model was developed which demonstrated a relationship between resistivity and the relative volume fraction of the binder material separating the conductive particles where higher curing temperatures lowered the amount of binder in the ink system and conductivity increased. For nanoparticle inks, the full microstructural evolution which occurs during thermal processing has also been well characterized in which sintering is the mechanism for creating a semisolid film [13,14,15]. In this case, semisolid films can be created after curing at relatively low temperatures (<300 °C) due to the effect of particle size on melting temperature. The relationship between particle size and melting temperature has been modeled for Au [16] and Ag [17,18] nanoparticles and a depression of melting temperature is observed when the particle size is at or below the nano-regime. The relationship between size and physical properties is sometimes referred to as the “scaling law” [19] and many examples exist in nanoscale literature.

The allowable curing temperature range is largely dictated by the substrate choice, which is in turn dependent on the particular electronics application. For example, applications requiring flexible substrates could employ the use of a polymeric material as a substrate. The glass transition temperature of polymers in many cases will overlap with or be below the manufacturer recommended curing parameters. On the other hand, an application in which flexibility is not needed could use a rigid substrate, such as ceramic, which can withstand temperatures higher than the melting point of the printed conductor. The ability to cure the printed conductor near the melting temperature of the conductive particles allows the electrical performance of these conductors to be improved dramatically. Understanding the effect of microstructure on electrical performance is important when selecting a conductive ink for utilizing printing technologies in the production of electronic systems especially when polymeric substrates are used due to the corresponding temperature limits. In this paper, a comparison is made between micro and nanoparticle loaded conductive inks. The microstructural changes which occur as a result of thermally curing these two ink types are documented. Two printing techniques are utilized, inkjetting of nanoparticle loaded inks and direct write micro dispensing of microparticle loaded inks.

## 2. Experimental

### 2.1. Ag Microparticle Inks on Flexible and Rigid Substrates

For the characterization of microparticle inks, two substrates were used. The first substrate was a flexible DuPont Kapton^®^ substrate (DuPont, Wilmington, DE, USA) while the second was a rigid ceramic 96% alumina substrate. The ceramic substrate has the benefit of being able to withstand high temperatures, and these high temperatures substantially improve the electrical conductivity of the ink by permitting particle sintering. Conversely, the Kapton^®^ substrate has the desirable property of being flexible, which allows conductive lines printed on this substrate to be used in conformal or flexible applications, and in general, increases the application potential. However, the drawback of Kapton^®^ is that thermal processing is typically restricted to temperatures below the glass transition temperature of approximately 360 °C [20], around 100 °C below that required for the sintering of Ag particles of sizes in the micro regime.

**Figure 1:**
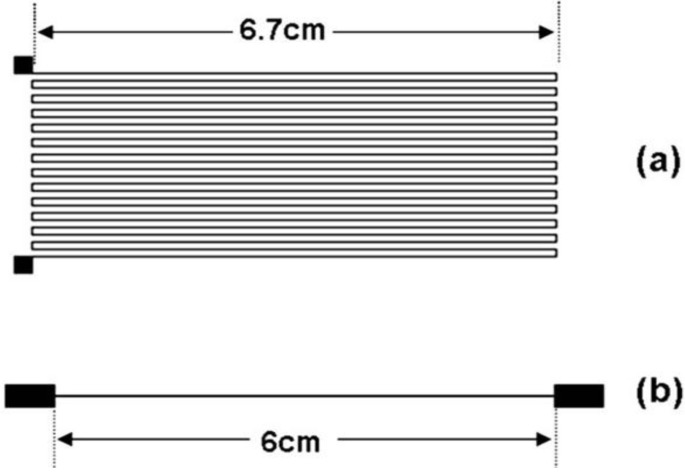
The **(a)** serpentine pattern and **(b)** straight line pattern used in the testing of Ag particle loaded inks. The target line width for microparticle inks was 250 μm while the target line width for nanoparticle inks was 850 μm.

For experiments involving microparticle inks, printing was performed via the direct write micro dispensing technique with an nScrypt 3D 450 system (nScrypt, Inc., Orlando, Florida, USA). The patterns shown in Figure 1 were printed for the experiments. Table 1 summarizes the experimental parameters for all inks used in this paper. The first ink type was Ferro 3309F conductive ink (Ferro Corporation, Cleveland, OH, USA) which was designed for use on alumina ceramic substrates and has a recommended maximum cure temperature of 850 °C [21] as used in commercial Low Temperature Co-Fired Ceramics (LTCC) technology. The Ferro 3309F ink was printed on a rigid ceramic 96% alumina substrate in the pattern seen in Figure 1(a) and then subjected to a curing cycle starting at 110 °C for 20 min and then increasing the temperature to 850 °C and maintaining the temperature at 850 °C for 10 min. The total cure cycle, including the temperature ramp, lasted 2 h.

**Table 1:** The inks, print methods, substrates, test patterns, and curing parameters for experiments performed in this study.

Ink	Print Method	Substrate	Test Pattern	Cure Parameters
**Ferro 3309F**	Direct write	Alumina	Serpentine	Cycled to 850 °C max
		Kapton^®^	Serpentine	160 °C 1 h
**DuPont CB028**	Direct write	Kapton^®^	Serpentine	160 °C 1 h
**Ercon E1660**	Direct write	Kapton^®^	Line	Air Dried
				138 °C 1h
**Cabot CCI-300**	Inkjet	Kapton^®^	Line	100 °C 1 h
			Line	110 °C 1 h
			Line	150 °C 1 h
			Line	160 °C 1 h
			Line	175 °C 1 h
			Line	200 °C 1 h
			Line	250 °C 1 h, 24 h

The second conductive ink used, DuPont CB028 (DuPont, Wilmington, DE, USA), has a lower recommended curing temperature of 160 °C [22] and is therefore often targeted for use in flexible applications that require a low maximum processing temperature. The DuPont CB028 ink was printed on DuPont Kapton^®^ polyimide substrates in the pattern displayed in Figure 1(a) and then thermally cured at 160 °C for 1 h. To further compare the effects of processing temperature, a second set of Ferro 3309F was printed on the same type of polyimide substrate and cured at 160 °C for 1 h, although this is a significant departure from the manufacturer’s recommendation.

**Figure 2:**
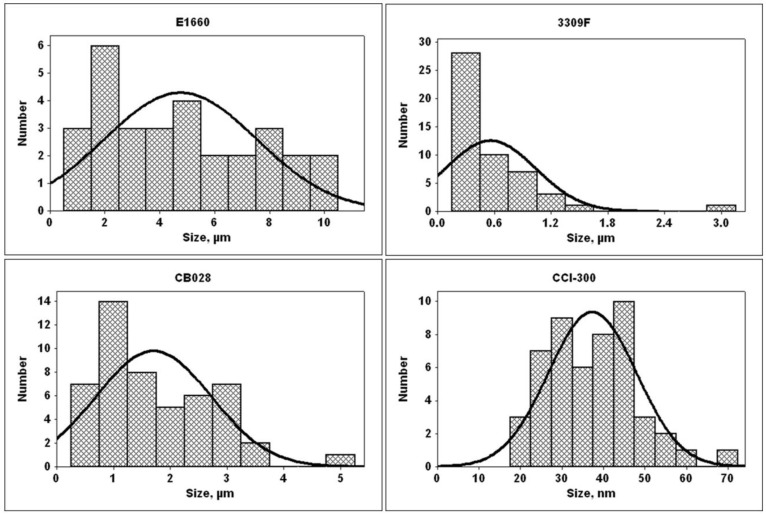
Measured size distributions of the conductive particles used in all experiments. Measurements were made from SEM micrographs.

A third microparticle loaded ink, Ercon E1660 (Ercon Incorporated, Wareham, MA USA), was printed via direct write micro dispense on a Kapton^®^ substrate in the pattern depicted in Figure 1(b). Ercon E1660 is designed for use on both rigid and flexible substrates and has a recommended curing temperature of 121 °C [23]. Two cure methods were employed: (1) Air drying and (2) Curing at 138 °C for 1 h. The temperature of 138 °C was utilized based on a previous performance characterization of this ink by Navarrete *et al.* [24]. The size distribution of the particles within the ink was measured from SEM micrographs for the Ercon E1660 as well as the first two inks discussed previously. As seen in Figure 2, the E1660 is loaded with Ag particles, which are roughly twice as large as the particles in the DuPont ink and roughly 10 times as large as the particles in the Ferro ink.

Resistivity of the printed traces was calculated based on dimensional measurements of the printed patterns and the measured resistance. The equation used to calculate resistivity, *ρ*, is

(1) $$\rho =R\frac{A}{l}$$

where *R* is measured resistance, *A* is the cross-sectional area of the printed line, and *l* is the length of the printed line. For each ink and substrate combination, multiple samples were printed and the resistance was measured using a Keithley Model 2000 multimeter (Keithley Instruments, Inc., Cleveland, OH, USA). Cross-sectional areas of the printed lines were calculated by first measuring the line thickness with a KLA-Tencor Alpha Step IQ stylus profilometer (KLA-Tencor, Milpitas, CA, USA.) operating with a resolution of 20 nm. An outline representing the cross section of the printed line was generated by plotting x *vs*. z where the thickness was taken to be along the z-axis and the width to be along the x-axis. The cross-sectional area was approximated by calculating the area beneath the plotted curve utilizing a Riemann sum-type approach

(2) $$Area=\sum_{i=1}^{n}z_{i}\Delta x_{i}$$

**Figure 3:**
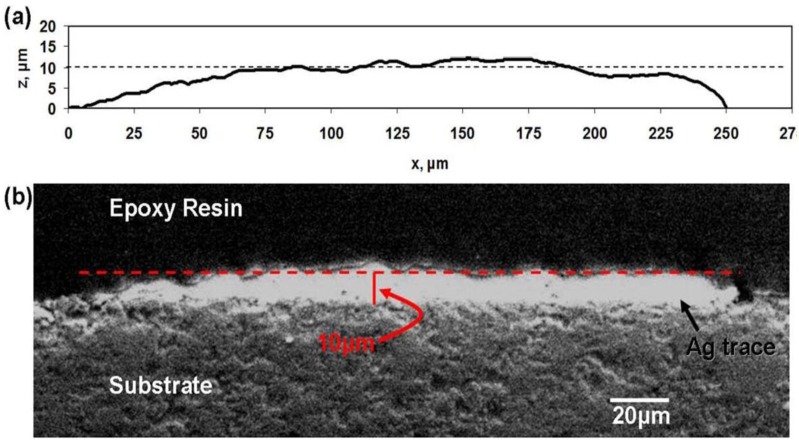
**(a)** Example of a randomly measured profile of a printed trace of Ferro 3309F on a ceramic substrate and **(b)** SEM micrograph of a random cross section of a printed trace of Ferro 3309F on a ceramic substrate.

In this case, ∆x is 1 μm. An example of the graphical representation of the cross section of a printed line is seen in Figure 3. Validation of the profilometer method was made by examining SEM cross sections of a printed line of Ferro 3309F on an alumina substrate and comparing the thickness of the printed line to the measured profile as seen in Figure 3. Resistivity was then calculated based on the known length of 1.8 m for the serpentine patterns and 6 cm for the straight line patterns seen in Figure 1(a) and (b) respectively. Comparing random measurements among all of the printed samples revealed a variation in measured cross section to be roughly 10%.

Specimen preparation for scanning electron microscope (SEM) microanalysis of the printed lines entailed the use of a surgical scalpel to section the polymeric substrates and the use of a glass cutter to section the ceramic substrates. The samples were then subjected to Au sputtering for 30 s with a Gatan Model 682 Precision Etching Coating System (Gatan, Inc., Pleasanton, CA, USA) to reduce charge effects in the SEM. SEM was performed on all samples in a top down fashion using a Hitachi S-4800 Ultra-high Resolution Field Emission Scanning Electron Microscope (Hitachi High-Technologies Corporation, Tokyo, Japan) utilizing a 20 keV accelerating voltage.

### 2.2. Ag Nanoparticle Ink on Flexible Substrates

An alternative to using microparticle-based inks in applications where the cure process is controlled by the thermal constraints of a polymeric substrate is to use a nanoparticle-based ink and take advantage of the reduced sintering temperature found in the nano regime. In order to explore this option, the 6 cm long pattern seen in Figure 1(b) was utilized in the characterization of a commercially available Ag nanoparticle ink, Cabot CCI-300, (Cabot Corporation, Albuquerque, NM, USA). The test pattern was inkjet printed on a Kapton^®^ substrate with a Dimatix 2800-Series Materials Printer (Fujifilm Dimatix, Inc, Santa Clara, CA, USA). One layer (single print) was printed at 1270 DPI. In order to improve the wetting characteristics of the ink substrate interface, the Kapton^®^ substrate was first cleaned with UV-Ozone for 10 min with a Jelight UVO Cleaner Model 342A (Jelight Company, Inc, Irvine, CA, USA). Sample sets of five were oven cured for 1 h at each of the following temperatures: 100 °C, 110 °C, 150 °C, 160 °C, 175 °C, 200 °C, and 250 °C. SEM microanalysis in a top down fashion was performed as described previously. Resistivity was determined based on the technique outlined previously for microparticle inks.

## 3. Results and Discussion

### 3.1. Ag Microparticle Inks on Flexible and Rigid Substrates

**Figure 4:**
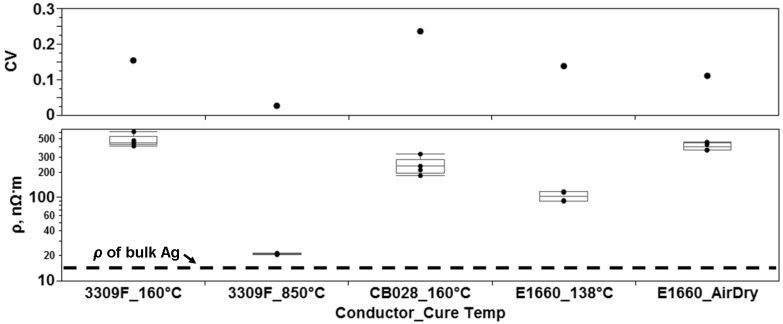
Graphical results of the measured resistivity for the thermal cure experiments involving flake-based inks. Note the variation of the sample set is greater for patterns with a higher resistivity.

Using the resistance and dimensional measurements of the printed lines, resistivity for the inks was calculated and the graphical results are seen in Figure 4. Comparing the measured results to the bulk resistivity of Ag, 14.6 nΩ•m [25], allows for the results of the cure experiments to be benchmarked. The Ferro 3309F patterns cured at 850 °C on ceramic substrates produced resistivity values calculated to be 21 nΩ•m +/−5.7 nΩ•m while the resistivity for the patterns printed on Kapton^®^ using the same ink cured at 160 °C was calculated to be 480 nΩ•m +/−74 nΩ•m. The resistivity of the DuPont CB028 ink samples printed on Kapton^®^ and cured at 160 °C was calculated to be 239 nΩ•m +/−57 nΩ•m. The Ercon E1660 samples in the air dried condition had a resistivity of 405 nΩ•m +/−45 nΩ•m while samples of the same ink cured at 138 °C for 1h had a resistivity of 103 nΩ•m +/−14 nΩ•m. The three inks printed on Kapton^®^ were roughly an order of magnitude less conductive than the patterns printed on ceramic due to the ability of the ceramic substrate to withstand the higher curing temperature. The DuPont ink cured at 160 °C is roughly two times more conductive than the Ferro ink cured at the same temperature. Utilizing the Ferro 3309F processed at this temperature is not the manufacturer intended application as the Ferro 3309F ink was optimized for 850 °C. The air drying of the E1660 samples produced a resistivity on par with the 3309F cured at 160 °C while the Ercon E1660 cured at 138 °C produced the most conductive trace for the inks printed on Kapton^®^. This significantly better performance is potentially due to the average particle size being the largest among the inks tested. The implication made here is that percolation theory should also be characterized in terms of particle size in addition to the amount of binder material separating the conductive particles. However, the larger particle size restricts the minimum feature size that can be printed.

Based on the comparison of the measured results to the bulk resistivity of Ag, a conclusion can be drawn that the Ferro 3309F printed on ceramic has created a conductive path with a conductivity approaching bulk Ag (67% of bulk Ag) due to the higher temperatures involved in the curing process. A conclusion can also be drawn that as the Ferro is intended for LTCC processing which will create a microstructure favorable to conductivity—in this case, grains.

**Figure 5:**
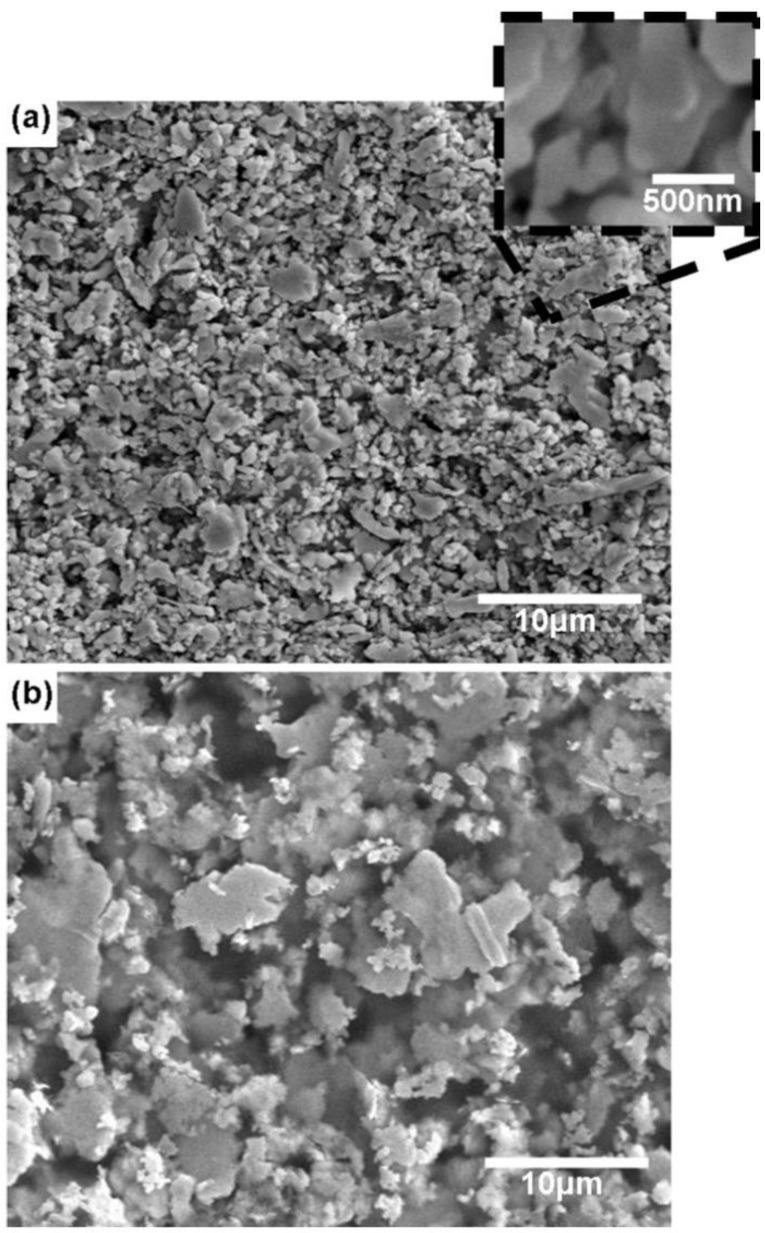
**(a)** SEM micrograph of DuPont CB028 ink after thermally curing for 1 h at 160 °C and **(b)** Ercon E1660 after thermally curing for 1 h at 138 °C.

**Figure 6:**
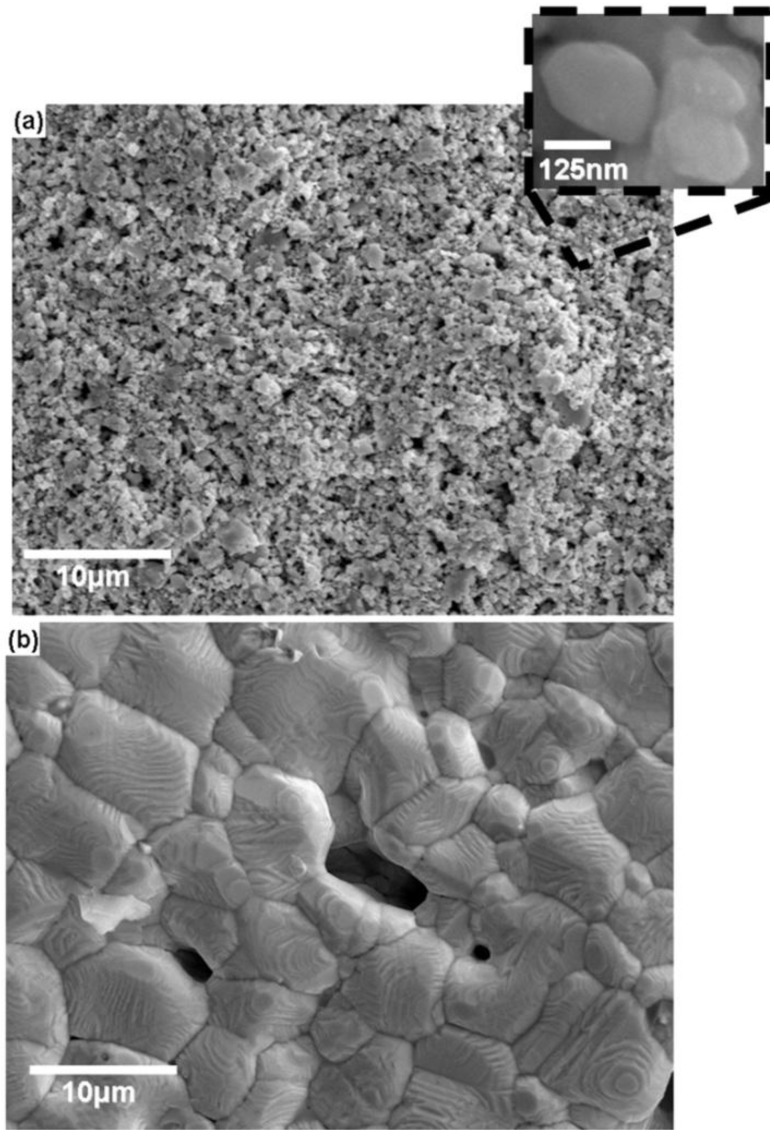
SEM Micrographs of Ferro 3309F **(a)** after thermally curing for 1 h at 160 °C and **(b)** after a 2 h thermal cure cycle which peaked at 850 °C for 10 min. Note the extreme difference in microstructure.

Microstructural characterization reveals differences in the governing factors controlling conductivity, namely conduction through physical contact between particles vs. conduction in a grainy film. In the case of both DuPont CB028 and Ercon E1660, the microstructure is that of individual particles conducting as a result of the particles physically touching one another as seen in Figure 5. The decrease in resistivity between air dried samples and thermally cured samples of both DuPont CB028 and Ercon E1660 is in agreement with the results noted by Saraf *et al.* [12] which showed an increase in conductivity for higher curing temperatures compared to lower curing temperatures for a microparticle based ink. In the case of Ferro 3309F, the thermal processing at 850 °C changed the microstructure of the ink printed on ceramic to a semi-porous film as seen in Figure 6(b), with a grain diameter of ~5 μm as determined by the linear intercept method. The differences between both high and low temperature curing of the Ferro 3309F ink are clearly illustrated in Figure 6. After low temperature curing, the morphology is that of slightly sintered particles mixed with larger particles as seen in Figure 6(a). The key aspect of this microstructure is particles and sintered groups of particles touching each other. Conversely, after the high temperature curing, the transformation from particles of a sub-micron size to a near solid form composed of faceted grains on the order of ~5 μm in diameter is indicative of a solid state grain growth process via grain boundary diffusion, a process well-characterized in the sintering of Ag and other materials [26,27]. The ability of the alumina ceramic substrate to withstand the temperature required to initiate this solid state growth process has enabled the transformation from particle to a near-solid porous film and consequently, these films provide the best conductivity which is approaching that of bulk Ag—14.6 nΩ•m [25]

The impact of grain size and porosity on the resistivity of a printed conductive trace has been characterized in literature [28,29]. The key aspects of these models are that microstructures consisting of large grains are more conductive than microstructures consisting of small grains and that less porosity is more favorable to conductivity. Consequently, the resistivity of the Ferro 3309F ink processed at higher temperatures is governed by the grain size and the amount of pores in the film. By contrast, the lower temperature thermal processing of the DuPont CB028, Ercon E1660, and Ferro3309F inks printed on Kapton^®^ did not result in the creation of a film and therefore the resistivity is higher due to the dependence of resistivity on the amount of physical contact between the Ag particles. The difference between the two main factors controlling resistivity (contact area *vs*. grain size) is graphically represented in Figure 4. A difference exists not only in resistivity between the printed lines cured at high and low temperatures, but also in the distribution of the data. Figure 4 illustrates a higher variability (defined by the coefficient of variation, CV) associated with the previously mentioned percolation theory mechanism.

### 3.2. Ag Nanoparticle Loaded Conductor on Flexible Substrate

**Figure 7:**
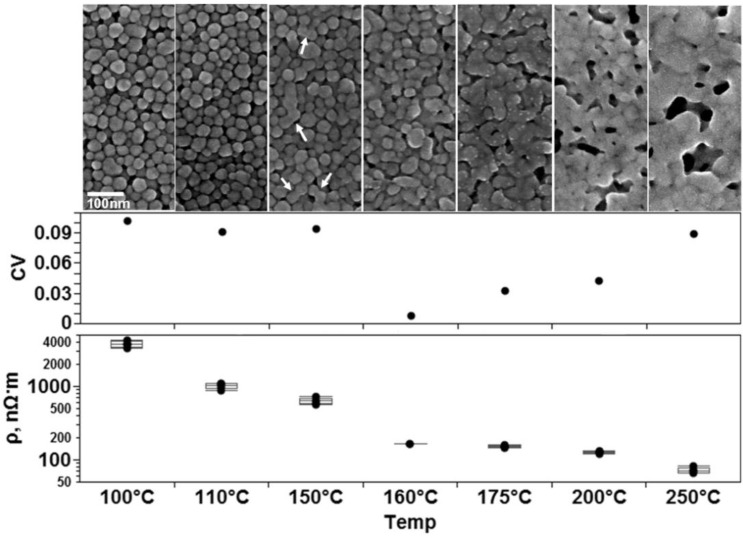
SEM micrographs of the microstructures resulting from thermally curing printed traces of Cabot CCI-300 conductive ink for 1 h at the indicated temperatures. All images are at the same magnification. The corresponding graphical results of measured resistivity (ρ) are below the micrographs. The relationship between variation and method of conduction is not as clear as in the case of microparticle based ink. Sample size n = 5 for all sets.

The microstructural evolution, which occurs with an increase in processing temperature, is illustrated in Figure 7 for the Cabot CCI-300 lines printed on Kapton^®^ substrates. The graphical results of the corresponding resistivity (ρ) measurements for each curing temperature are also seen in Figure 7. Curing temperatures of 100 °C and 110 °C resulted in a microstructure consisting of unaltered Ag particles. The method of conduction at these two temperatures is percolation and it is assumed that curing at 110 °C removed more solvent, which increased the amount of contact between the particles and thus, the conductivity. The assumption based on the observed decrease in resistivity is consistent with the previously mentioned percolation theory and the results noted in [12] and [13]. At 150 °C the microstructure is that of slightly necked particles—examples of which are indicated by white arrows. At 160 °C and 175 °C the sintering process is more pronounced as indicated by the neck growth. A relationship between conductivity and the neck diameter of sintering particles has been documented in [13] where conductivity increases as the neck diameter grows. Curing at 200 °C and 250 °C resulted in a film-like microstructure consisting of fully sintered particles and voids which can only occur at these processing temperatures.

**Figure 8:**
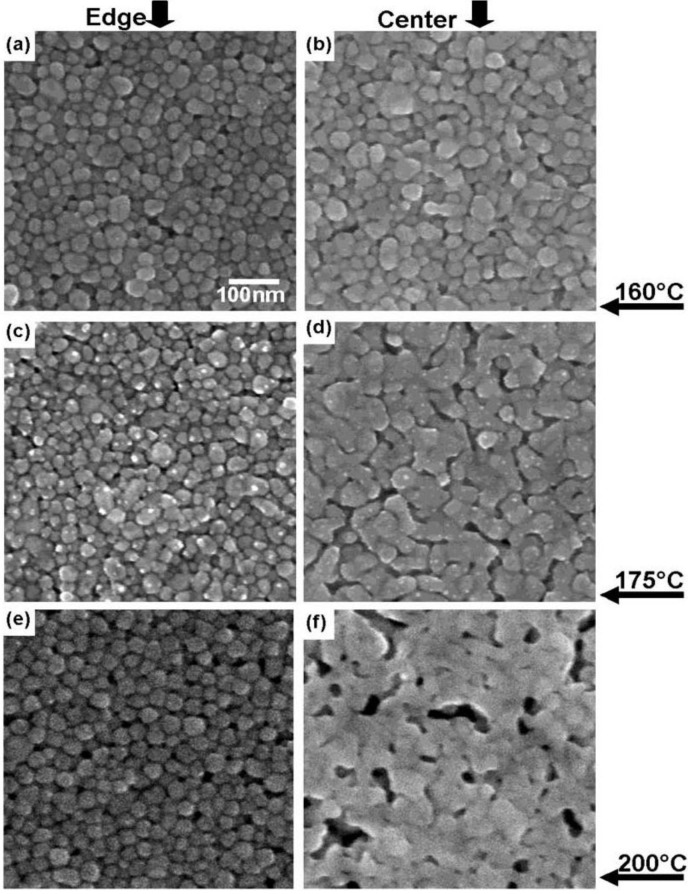
SEM micrographs of CCI-300 printed lines comparing **(a)** the edge of a printed line with **(b)** the center of a printed line cured at 160 °C, **(c)** the edge and **(d)** the center of a line cured at 175 °C, **(e)** the edge and **(f)** the center of a line cured at 200 °C. All lines were printed in one layer and cured for 1 h. All images are the same magnification.

Analyzing the variability in resistance measurements of the nanoparticle loaded ink does not reveal as clear of a relationship between method of conduction and variability as the results of the microparticle loaded inks did. One reason for this may be the non-homogenous nature of the inkjet printing process. When comparing the microstructure of the edge of the printed line with the center of the printed line, a difference between the degree of sintering is observed as seen in Figure 8. One reason for this occurrence may be due to the particles being less densely packed on the edge of the printed line. Sintering is governed by various diffusion processes beginning with surface diffusion [13] which has to rely on the amount of contact between the particles.

**Figure 9:**
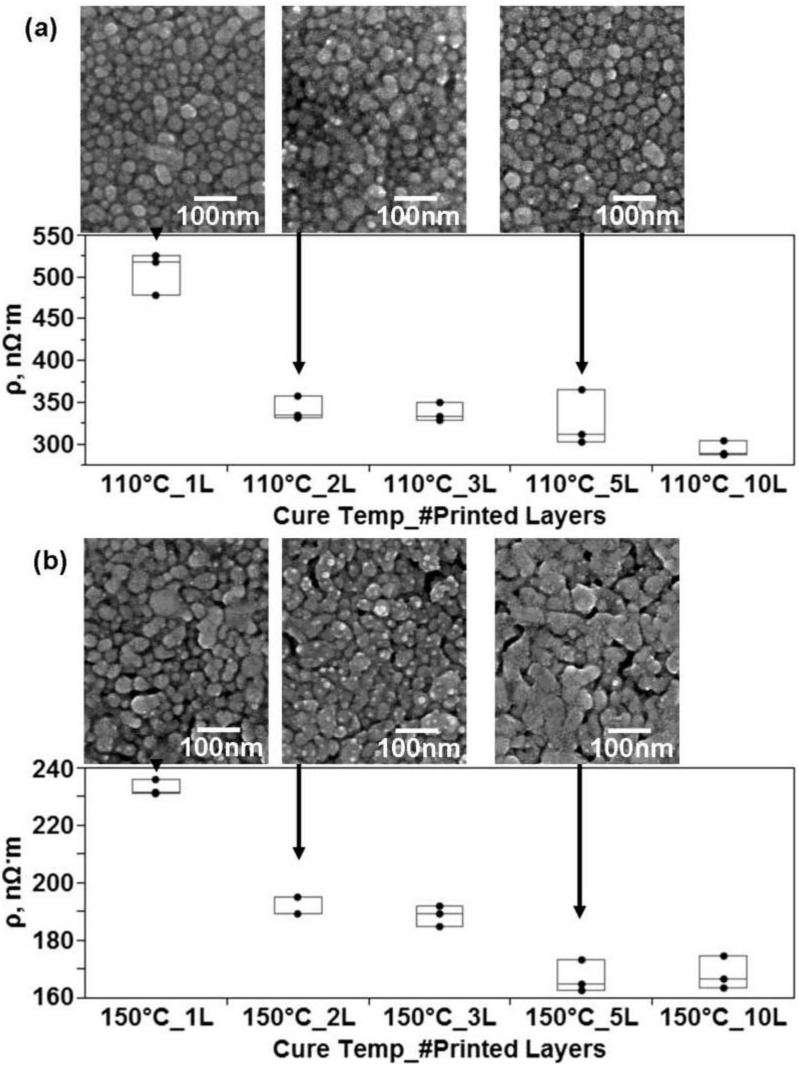
Comparison of the microstructures and resistivity measurements of printed lines of CCI-300 resulting form a difference in the amount of layers printed prior to printing for **(a)** lines cured for 1 h at 110 °C and **(b)** lines cured for 1 h at 150 °C. Note the drop in resistivity when the amount of layers is increased to 2.

To further explore the effect of particle contact area on the resulting microstructure, printing the pattern in multiple times was explored. Printing the same pattern with two or more layers—essentially printing the pattern multiple times on itself—results in a decrease in resistivity though expectation is that resistivity should be the same between one and multiple layers as the ratio of area over length should normalize the measurements. All samples were printed at the same time and then thermally cured at the same time for the given cure temperature. Analyzing the microstructural differences between lines printed in one and multiple layers may explain the drop in resistivity. Comparing the microstructures (Figure 9) of lines printed in one layer with lines printed in multiple layers and then cured at 110 °C shows the microstructure to remain largely the same between printing in one, two and five layers. Examining the microstructures of lines printed at 150 °C for 1 h reveals the sintering process to be enhanced as a function of the number of layers printed.

The fact the necking process is more evolved at the same processing temperature is consistent with the notion of the dense packing model controlling the amount of sintering and also follows observations made on the edge of the printed lines in the previous experiment. Also seen in Figure 9 is a graphical representation of the effect of increasing the number of printed layers on the resistivity. It is notable here that the resistivity of lines cured at 110 °C and 150 °C decreases by a factor of 1.2 and 1.5 respectively between one and two layers of printing. The conclusion can be made that printing more layers results in a higher amount of contact between particles. The basis for this reasoning is that two phenomena are enhanced when increasing the amount of printed layers. The first phenomenon is conduction through percolation which increases as the amount of contact area grows. The increase in conductivity for lines cured at 110 °C can only be explained by greater contact area between particles. Also governed by contact area are the diffusion mechanisms driving neck growth which was enhanced by increasing the number of printed layers for lines cured at 150 °C as seen in Figure 9.

**Figure 10:**
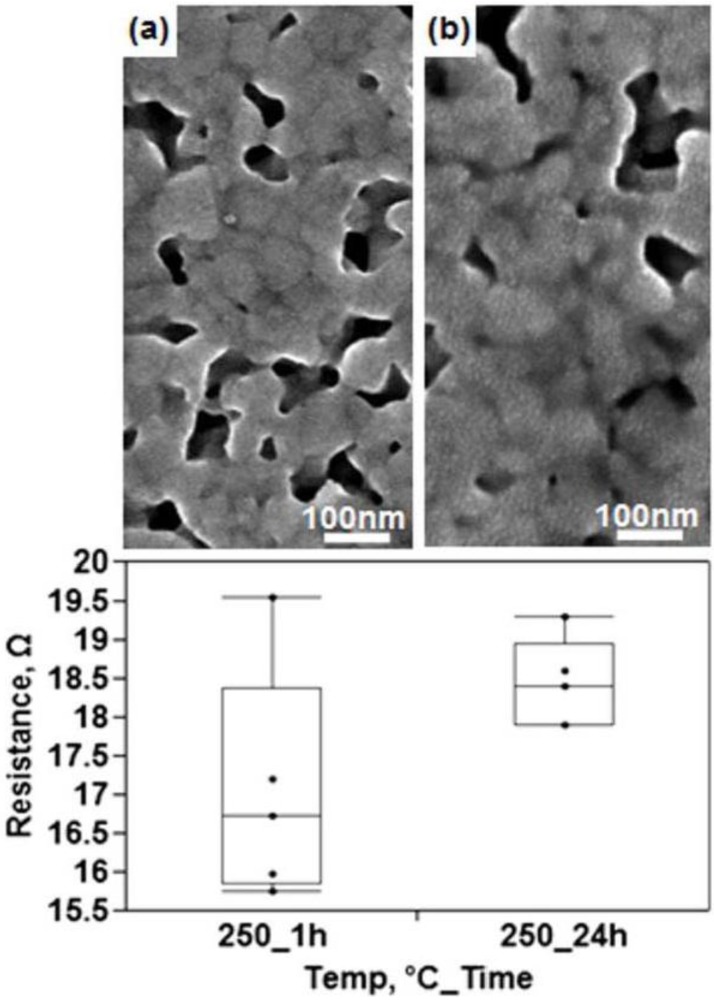
SEM micrographs of Cabot CCI-300 cured at 250 °C for **(a)** 1 h and **(b)** 24 h along with corresponding graph of resistance measurements.

Comparing the microstructure of the fully sintered film to that of the Ferro 3309F ink cured at 850 °C reveals limitations of utilizing nanoparticle ink. Despite being fully sintered at 250 °C, there is no grain growth occurring at this processing temperature. Increasing the cure time to 24 h did not result in grain coarsening as is illustrated in Figure 10. Conversely, the higher thermal processing of the conductive ink printed on ceramic substrates resulted in coarse faceted grains. The grainy microstructure is pertinent to electrical performance due to the previously discussed relationship between grain size and resistivity. Though the capability of the nanoparticles to sinter at a depressed temperature offers the ability to process at a relatively low temperature, once the sintering process is complete, the resultant film would need a higher temperature processing for the creation of coarse grains to occur. A general rule of thumb for temperatures needed to induce grain growth in metals is roughly half the melting temperature of the given metal. In the case of silver, a temperature of around 460 °C would be required to coarsen the grains. Furthermore, longer curing times appear to have an adverse effect on the electrical performance in terms of measured resistance as seen in Figure 10 that is potentially the result of oxidation as the thermal curing did not take place in an inert environment. According to the manufacturer specification sheet for Cabot CCI-300 ink [30], the maximum cure temperature is 350 °C, however electrical characterization data on the same data sheet showed no improvement to electrical resistivity for curing temperatures above 250 °C.

**Figure 11:**
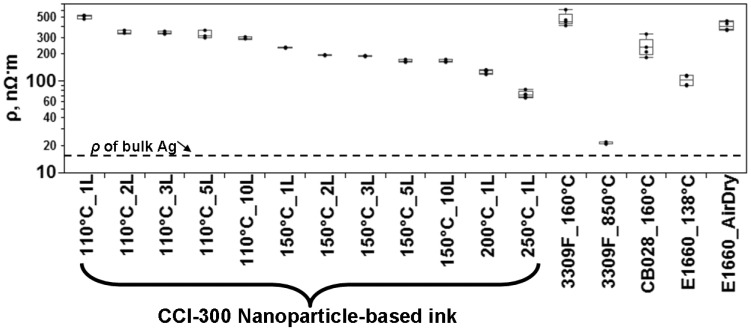
Comparison of resistivity (ρ) measurements for experiments conducted in this study.

Analysis of the data from all the experiments performed in this study reveals two trends. The first trend was observed from examining the variability of the resistivity of the printed samples. As the microstructure becomes more conductive, there is less variability. In the case of microparticle loaded inks, conduction through percolation yields a more variable situation than conduction through grainy film. The trend of a decrease in variability as the resistivity lowers is also observable in the processing of printed lines composed of nanoparticle loaded ink though not as pronounced as the trend observed in the microparticle loaded ink experiments. The second observable trend is a decrease in resistivity in inkjet printed lines when the pattern is printed two or more times prior to the application of the thermal curing process. Figure 11 is a graphical representation of the resistivity for some experiments in this study and allows for a comparison between the electrical performance of microparticle and nanoparticle loaded inks.

When deciding which ink type to use, the electrical performance is only one parameter to consider along with the recommended curing temperature. From the graph in Figure 11, the electrical performance in terms of resistivity of the Ercon E1660 is comparable to the nanoparticle loaded ink tested in this study. However, in terms of the micro (or nano) constituents of the ink, the minimum feature size is limited by the large particle size within the ink—as large as 10 μm in diameter according to the measured size distribution in Figure 2. Obviously, if submicron features are desired, the ink utilized would have to be composed of submicron particles though a printing technique other than inkjet would be needed as the minimum feature size of this process is greater than 1 μm [31,32].

## 4. Conclusions

The microstructures, which arise as a result of the thermal processing of Ag particle loaded inks, play a role in the overall electrical performance of printed conductive traces. The key enabler for the most conductive microstructure observed in the experiments performed in this paper was high temperature processing (greater than half the melting temperature of the conductive particles) of the printed traces. The thermal processing in this temperature regime can only be withstood by rigid ceramic substrates. The dominant characteristic of this microstructure are grains of 5 μm in diameter.

An observable trend in the decrease in sample set variability with an increase in conductivity exists in the processing of microparticle loaded conductors that corresponds with a microstructural change incurred by the thermal processing of printed traces. The trend is not as clear when examining the resistivity of lines created from the inkjet printing of nanoparticle loaded ink due to the lack of homogeneity of the microstructure at the edge of the printed lines.

Despite the fact lines created via inkjet printing of Ag nanoparticle loaded ink have the ability to attain a more conductive microstructure due to the ability of the nanoparticles to undergo sintering at low temperatures, lines created with Ag microparticle based inks (particles on the order of 5 μm) will have a similar resistivity. In the case of nanoparticle loaded inks, once a sintered film is attained, temperatures on the order of 460 °C would be required to obtain a coarse grain structure.

A profound nuance of inkjet printed traces is that the number of layers printed before curing results in a decrease in the measured resistivity of the conductive trace. The reduction in resistivity may be due to the densification of the packing of the nanoparticles resulting from the additional of layers. The densification results in a higher contact area between the individual particles which will enhance conduction through percolation when sintering has not occurred. The sintering process is enhanced by increasing the amount of layers printed which may also be due to more contact between the Ag nanoparticles which would enhance the diffusion mechanisms driving the sintering process.
